# Eyelid Carcinoma in Patients with Systemic Lymphoma

**Published:** 2010-01

**Authors:** Debraj Shome, Diana Bell, Bita Esmaeli

**Affiliations:** M. D. Anderson Cancer Center, University of Texas, Houston, Texas, USA

**Keywords:** Systemic Lymphoma, Immunosuppression, Eyelid Cancer, Squamous Cell Carcinoma

## Abstract

**Purpose:**

To describe a series of patients with Non-Hodgkin’s lymphoma (NHL) and concomitant eyelid carcinoma.

**Methods:**

In this non-comparative interventional case series, we retrospectively reviewed the medical records of 5 patients with NHL who developed eyelid carcinoma.

**Results:**

The patients included one female and four male subjects. Systemic lymphoma had been diagnosed 1 to 72 months prior to development of the eyelid carcinoma. The lesions were basal cell carcinoma in three, and squamous cell carcinoma in two cases. The lymphoma was advanced (stage III or IV) in all patients. Four patients underwent surgical excision of the carcinoma and one patient was awaiting surgical treatment after completing systemic chemotherapy. Three subjects had high-grade carcinomas. Two patients had perineural invasion; one received adjuvant radiotherapy postoperatively but the other did not due to receiving systemic chemotherapy for recurrent NHL.

**Conclusions:**

Systemic lymphoma may be associated with aggressive eyelid carcinomas. Perineural invasion is frequently encountered in this situation and should be treated with adjuvant radiation therapy to decrease the likelihood of local recurrence.

## INTRODUCTION

The incidence of non-Hodgkin’s lymphoma (NHL) is estimated to be rising 4% per year and to have doubled over the last two decades, especially in the industrialized world.^1^,^2^ The literature suggests an increased risk of other malignancies in patients with NHL and chronic lymphocytic leukemia (CLL). Specifically, lung carcinoma, brain cancer, ocular melanoma, Hodgkin disease, and melanoma and non-melanoma skin cancers are over-represented among patients with NHL or CLL.^3^–^6^ In patients with NHL or CLL, skin cancer has not only an increased incidence but also a more aggressive behaviour.^7^ An increased propensity for and aggressive nature of skin cancers^8^ and more specifically eyelid cancers,^9^,^10^ has been reported in states of systemic immunosuppression, such as solid organ transplantation. A Medline search did not reveal any report of eyelid carcinoma in patients with systemic NHL. Herein, we report five patients with systemic NHL who developed eyelid skin carcinomas and present a brief review on this topic.

## RESULTS

The patients included four male and one female Caucasian subjects who had NHL and were treated between June 2001 and June 2007. They developed eyelid carcinoma from 1 to 72 months after a diagnosis of systemic lymphoma. Table 1 summarizes patients’ data.

### Patient 1

A 61-year-old man presented with a lesion involving the central left lower eyelid margin. He had been diagnosed with mantle cell lymphoma 6 years prior to this presentation. The patient was initially treated with rituximab and cyclophosphamide, doxorubicin, vincristine and prednisone (R-CHOP). However, upon recurrence, the patient received unrelated donor stem cell transplantation, 13 months prior to appearance of the eyelid mass. His post-transplant course had been complicated by recurrent viral infections, including influenza and cytomegalovirus (CMV) reactivation. He had developed basal cell carcinoma (BCC) in his forehead and chin three years ago and was treated with surgical excision. Pertinent physical findings consisted of a central area of ulceration, with telangiectasia and crusting in the left lower eyelid. The patient was neutropenic at diagnosis (white blood cell count=4000/fL). Incisional biopsy of the mass revealed a squamous cell carcinoma (SCC) *in situ*. The patient underwent wide local excision of the mass with frozen section control of the margins. The defect was repaired using a tarsoconjunctival flap (Hughes procedure) from the left upper eyelid with a full thickness skin graft harvested from the left upper eyelid. The patient did well postoperatively with no evidence of local or regional recurrence at last contact, 5 months after surgery.

### Patient 2

A 61-year-old woman with a history of follicular NHL presented with a lesion in her left upper eyelid. Follicular lymphoma had been diagnosed three years ago and was being treated with chemotherapy (R-CHOP) at the time of presentation. She had a biopsy of the left upper eyelid lesion two years ago confirming BCC but the margins were not clear. When the patient presented to a Mohs surgeon, it was felt that the area of previous biopsy was undetectable and hence, further excision was deferred. The patient received no further intervention for this lesion and was under therapy for lymphoma in the interim. On examination, there was a well-circumscribed lesion at the upper eyelid margin. She was scheduled for surgical excision after completion of chemotherapy. The patient remains under close follow-up and there is only mild increase in the size of the lesion three months after initial presentation.

### Patient 3

**Figure 1:**
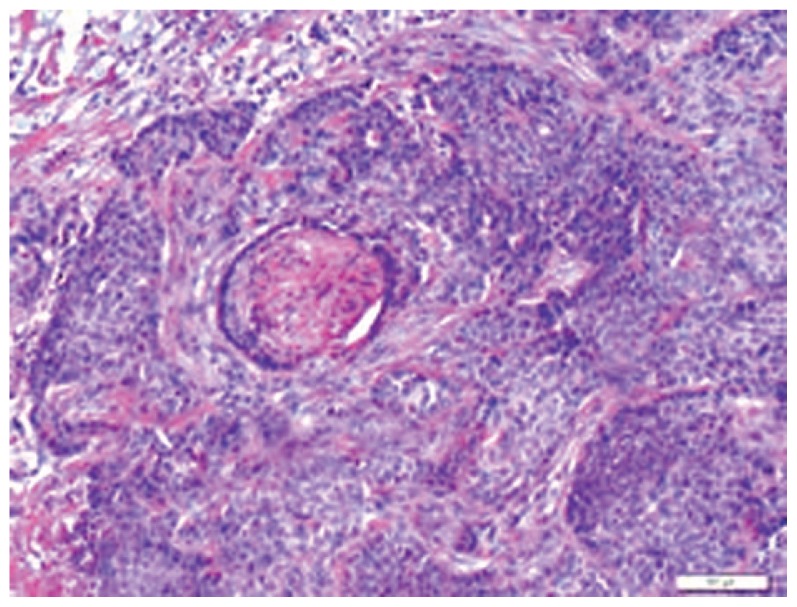
Histologic section of a basal cell carcinoma with perineural invasion (Hematoxylin and Eosin, 10×).

A 65-year-old man with a history of transformed follicular NHL was noted to have a mass in his right lateral canthus. The patient had stage IV NHL with bone marrow involvement, initially diagnosed one year ago. He had been treated with 6 courses of R-CHOP chemotherapy and had a complete response. He had first noticed a lesion in the right lateral canthus, 11 months prior to presentation to our clinic. Tumor resection by Mohs surgery was performed and the histologic diagnosis confirmed a BCC. Over the past 4 to 5 months, the patient has noted recurrence of the mass in the same area and the lesion had apparently almost doubled in size, in the month prior to presentation to our clinic. A recent biopsy showed BCC with a morpheaform (sclerosing) pattern and perineural invasion (Fig. 1).

**Figure 2:**
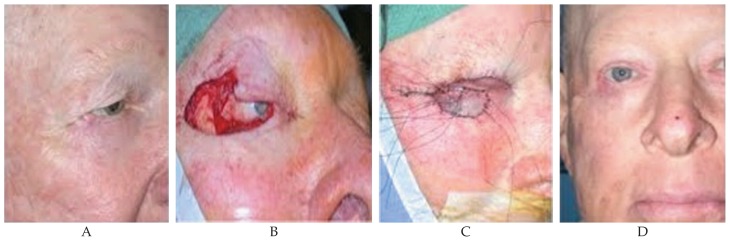
**A)** Basal cell carcinoma of the right lower eyelid and lateral canthus. **B)** The surgical defect after resection of the mass with frozen section control of the margins. **C)** The defect was closed using a tarsoconjunctival flap and a full-thickness skin graft. **D)** Final appearance after opening the flap and administration of postoperative adjuvant radiation therapy.

On examination, there was an ulcerative mass in the right lateral canthus (Fig. 2A). This mass was removed in its entirety, with 4 mm free margins. The excision included 2/3 of the lower eyelid, 1/3 of the upper eyelid and much of the soft tissue in the lateral canthus down to the orbital rim. A tissue sampling from the inner side of the orbital rim was also performed. Margin clearance was confirmed on frozen section and complex repair of the defect in the right upper eyelid was performed using a tarsoconjunctival flap (Figures 2B and 2C). Adjuvant postoperative radiotherapy was recommended given the extensive perineural invasion seen on the surgical specimen but this could not be carried out because the patient was simultaneously found to have recurrence of systemic lymphoma and required treatment with R-CHOP; therefore excessive dermal toxicity was expected from radiation therapy combined with Adriamycin. The patient is currently under close observation for recurrence of the carcinoma and radiation will be performed after cessation of the chemotherapy regimen for systemic lymphoma.

### Patient 4

A 63-year-old man with a history of stage III follicular NHL, presented with a poorly differentiated SCC of the left lower eyelid and cheek one year after a diagnosis of NHL. He had received R-CHOP for NHL, achieved complete remission and was still on maintenance Rituximab therapy when he developed the eyelid carcinoma. On examination, there was a lesion in the left lower eyelid and cheek in the distribution of the infraorbital nerve. A biopsy had revealed SCC with perineural invasion. The patient underwent surgical resection of this mass and infraorbital nerve dissection. He received postoperative adjuvant radiotherapy and has had no evidence of recurrence at last contact, 6 months after completion of radiotherapy.

### Patient 5

A 76-year-old man with a history of central nervous system lymphoma of large, diffuse, B-cell variety, and bilateral intraocular lymphoma, presented with a lesion in his lower eyelid and cheek that had been proven to be a BCC based on a recent biopsy. He had received systemic methotrexate and rituximab together with radiation therapy to both orbits, which had resulted in complete remission. On examination, there was an area of ulceration, with indurated margins, crusting and surrounding telangiectasia on the left lower eyelid, extending onto the cheek. Wide local excision of the lesion with frozen section control of the margins was performed. The patient has had no recurrence of eyelid carcinoma during 3 years of follow-up.

## DISCUSSION

We describe 5 patients with systemic lymphoma (mantle cell and follicular each in two, and diffuse large B-cell in one) who developed aggressive eyelid carcinoma (SCC in two and BCC in three patients). Lymphoma was advanced in all patients (stage III in two and stage IV in three). Three subjects had high-grade carcinomas and two had perineural invasion. Three cases had recurrent lesions after previous excision elsewhere. Eyelid cancers required complex reconstruction in four patients. In one patient (patient 4), a suprastructure maxillectomy with bone graft was needed in addition to eyelid reconstruction.

Otley^7^ analysed the highest-quality data examining the association between lymphoma and skin cancer, derived from population-based studies in Denmark, Sweden and Switzerland.^4^,^6^,^11^–^13^ These studies and others show a significantly increased risk for both melanoma and non-melanoma skin cancer among patients with NHL/CLL. The association between skin cancer and NHL or CLL is present when analysis is performed both on populations of patients with NHL or CLL, and those with skin cancer. The consistency of this association suggests a strong link. In studies that analyze the incidence of skin cancer by sex, both men and women with NHL or CLL experience increased rates. The association becomes stronger as the population becomes older.^7^

Multiple pathogenic mechanisms have been postulated to explain the association between skin cancer and NHL. Some authors have proposed that the association represents a causal link among sunlight damage, skin cancer and NHL. The evidence regarding the association of NHL/CLL with exposure to ultraviolet (UV) radiation is contradictory; however, the link between UV radiation and skin cancer is well established.^14^–^16^

Multiple hypotheses center on disease-related immunosuppression due to NHL as a cause of skin cancer, by which dysfunctional lymphocytes are unable to perform their usual immune surveillance and cytotoxic functions. This phenomenon most closely resembles immunosuppression related skin cancer in recipients of solid organ transplants.^17^ B cells have been shown to secrete immunosuppressive factors,^18^ and also to down-regulate CD40 ligand (CD154) expression on activated T cells, resulting in impaired interaction between activated T-cell with normal B cells and other antigen-presenting cells.^19^ Another hypothesis is that patients with NHL have an inherent susceptibility to other cancers, including skin cancer, through a genetic abnormality, HLA-associated variation, or environmental insults.^20^ This hypothesis has not been systematically explored.

Finally, standard therapy for NHL includes administration of cytotoxic chemotherapy which could serve as a mutagenic stimulus for subsequent development of cancer. These agents are also profoundly immunosuppressive. Chlorambucil and cyclophosphamide, both alkylating agents, have been associated with an increased risk of acute myeloid leukemia, and there are multiple reports of newer nucleoside analogues, such as fludarabine, being associated with secondary malignancies, including skin cancer.^20^–^22^ A synergistic effect of alkylating agents and nucleoside analogues on second malignancies has been surmised.

Skin cancers behave more aggressively in patients with NHL, in that they are associated with higher rates of recurrence, metastasis and even death.^23^–^25^ Our experience in patients with multiple recurrent eyelid carcinomas described in this series corroborates with this observation. We recommend meticulous control of surgical margins on frozen section at the time of surgical excision of eyelid carcinoma and administration of postoperative adjuvant radiation therapy if perineural invasion is encountered. The administration of postoperative adjuvant radiation therapy is expected to decrease the likelihood of local recurrence for eyelid carcinomas that harbor microscopic perineural invasion.^26^

## Figures and Tables

**Table 1:** Patients’ data

No	Age/Sex	Type of lymphoma, final stage	Therapy for systemic disease	Type of eyelid cancer	Time to first eyelid cancer (months)	Therapy for eyelid cancer	Follow-up (months)	Final systemic condition
1	61/M	Mantle Cell, IV	R-CHOP, BMT	SCC	72	Surgery	5	Alive, well
2	61/F	Follicular, III	R-CHOP	BCC	12	Observation	3	Alive, recurrence of lymphoma
3	65/M	Follicular, IV	R-CHOP	BCC	1	Surgery, RT recommended*	5	Alive, recurrence of lymphoma
4	63/M	Follicular, III A	R-CHOP	SCC	15	Surgery and post-op RT	6	Alive, well
5	76/M	DLBCL, IV	R-CHOP, RT (both orbits)	BCC	12	Surgery	36	Alive, well

M, Male; F, female; R-CHOP, Rituximab, Cytoxan, Hydroxydaunorubicin (Adriamycin), Oncovin (Vincristine), Prednisone/Prednisolone; BMT, bone marrow transplantation; SCC, squamous cell carcinoma; BCC, basal cell carcinoma; RT, radiation therapy; DLBCL, diffuse large B-cell lymphoma

* This patient had recurrence of lymphoma at the same time as recurrence of basal cell carcinoma with perineural invasion. Postoperative adjuvant radiotherapy could not be carried out due to the need to start R-CHOP immediately and due to concerns for excessive toxicity with the combination of the two treatments.
